# Unmasking the role of mast cells in dengue

**DOI:** 10.7554/eLife.00767

**Published:** 2013-04-30

**Authors:** Panisadee Avirutnan, Ponpan Matangkasombut

**Affiliations:** 1 **Panisadee Avirutnan** is at the Dengue Hemorrhagic Fever Research Unit, Faculty of Medicine Siriraj Hospital, Mahidol University, Bangkok, Thailandpanisadee.avi@mahidol.ac.th; 2 **Ponpan Matangkasombut** is at the Department of Microbiology, Faculty of Science, Mahidol University, Bangkok, Thailandponpan@post.harvard.edu

**Keywords:** mast cell, vascular leakage, dengue virus, chymase, leukotrienes, infectious disease, Human, Mouse, Viruses

## Abstract

Immune cells called mast cells can hinder rather than help the body's response to dengue virus, which suggests that mast cell products could be used as biomarkers to identify severe forms of the disease.

**Related research article** St John AL, Rathore APS, Raghavan B, Ng M-L, Abraham, SN. 2013. Contributions of mast cells and vasoactive products, leukotrienes and chymase, to dengue virus-induced vascular leakage. *eLife* **2**:e00481. doi: 10.7554/eLife.00481

**Image** Activated mast cells releasing their fluorescein-labeled granules (red) surround a blood vessel in tissue infected with dengue virus

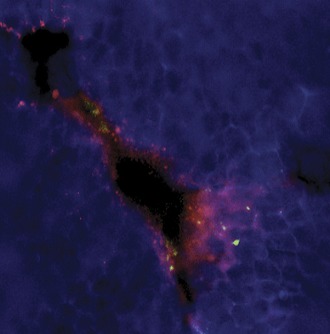

Every year, millions of people become infected with dengue virus: a mosquito-borne pathogen that poses an increasingly serious threat to global health. While the majority of individuals either experience no symptoms or mild dengue fever—a flu-like illness with headache, rash, and muscle and joint pain—a small percentage go on to develop a life-threatening condition called dengue hemorrhagic fever (DHF). This can result in hemorrhage, shock, organ failure and even death (World Health Organization, 1997).

DHF is marked by the leakage of plasma proteins and fluid out of capillaries so that the blood becomes more concentrated. This vascular leakage is accompanied by a decrease in the number of platelets—cell fragments that help the blood to clot—and abnormalities in liver function. At present, there is neither a vaccine nor any specific therapy for severe dengue infection, and progress on both these fronts has been hampered by an incomplete understanding of disease pathogenesis. Now, writing in *eLife*, Soman Abraham of Duke University and co-workers from the National University of Singapore and the Duke-NUS Graduate Medical School—including Ashley St John as first author—reveal a pathogenic role for immune cells called mast cells in the response to dengue infection (St John et al., 2013). The study also reveals a potential biomarker that could identify patients at risk of DHF, as well as novel therapeutic targets.

Mast cells are part of the innate immune system—the body’s first line of defense against pathogens—and reside in tissues surrounding blood vessels and lymphatic vessels. They can be activated by the binding of specific antibodies to receptors on their surface, and can also recognize pathogens and inflammatory proteins directly. Mast cells contain large numbers of granules, which are enriched in substances such as histamine, tryptase, chymase and tumor necrosis factor, and within seconds of the mast cells being activated, these substances are released in a process called degranulation. The activated mast cells also begin to synthesize leukotrienes, prostaglandins, cytokines, and other inflammatory mediators. The release of these substances/mediators increases the permeability of blood vessels and recruits immune cells to the site of the infection. It also leads to activation of various non-immune cells, such as smooth muscle cells and mucous glands, which help to remove allergens and pathogens from the body.

Several lines of evidence are consistent with a protective role for mast cells in the host response to pathogens (Abraham and St John, 2010). Dengue virus is known to infect mast cells, particularly in the presence of pre-existing antibodies from an earlier infection (King et al., 2000). Mast cells also contribute to immunosurveillance, responding to dengue virus by activating host anti-viral responses and releasing signaling molecules that recruit additional immune cells (St John et al., 2011). By contrast, other recent work suggests that mast cells may sometimes have a pathogenic role. When infected with dengue virus in vitro, they trigger activation of endothelial cells (Brown et al., 2011). These line the inside of blood vessels and their activation can promote inflammation and blood clotting. Moreover, levels of certain mast cell proteins are elevated in patients with DHF compared to those with milder dengue fever (Furuta et al., 2012).

To begin to decipher the pathogenic role of mast cells, St John et al. injected dengue virus under the skin of the mouse ear. There is some debate over whether normal mice are a suitable model for studying dengue virus infection as the virus does not replicate efficiently in mice, and the animals do not reproduce all of the symptoms seen in humans (Yauch and Shresta, 2008). However, local activation of mast cells (degranulation) and signs of capillary leakage were observed in the mice, which suggests that mice can be used to study dengue virus.

To delineate the role of mast cells in systemic dengue infection, the Duke-NUS team then introduced a clinical isolate of dengue virus directly into the mouse abdominal cavity. This induced a number of responses similar to those seen in patients with DHF, and which are consistent with increased vascular permeability. The mice also showed evidence of the virus in their bloodstream, as well as viral replication in the liver and spleen—the two primary organs affected in humans—and expressed a marker for mast cell activation, MCPT1. Similar findings were obtained in immunocompromised mice in which dengue virus can replicate more efficiently over a longer period. By contrast, genetically modified mice that were deficient in mast cells did not show vascular leakage in response to dengue virus, but began to do so when they were supplied with mast cells. Moreover, treatment with clinically approved mast-cell stabilizing compounds inhibited both mast cell activation and vascular leakage, in normal as well as immunocompromised mice.

To verify the mouse data, levels of chymase—the human equivalent of mouse MCPT1—were measured in a cohort of infected adults. The early clinical presentation of dengue fever and DHF are similar, but St John et al. found that chymase levels were consistently elevated in DHF patients compared to those with dengue fever. These findings point to a role for chymase (or mast cell activation) in the blood vessel pathology associated with dengue infection, and suggest that it could be a useful biomarker for DHF. Further studies are required to investigate the sensitivity, specificity, and predictive value of the test at various time points, and to determine whether it would also work in children.

The work of St John, Abraham and co-workers has revealed mast cells to be another deleterious player in dengue pathogenesis, despite their previously reported protective function. However, the factors that determine whether the mast cell response is beneficial or harmful are still unclear (Figure 1). The type and location of infected cells is likely to be important as different groups of mast cells can release distinct mediators. Whether the cells are activated by the virus itself or by pre-existing antibodies could also influence the response outcome, as could viral dosage. Lastly, the activity of other immune system components could influence the response of mast cells, as could the patient's genetic background and whether they have allergies.

**Figure 1.**
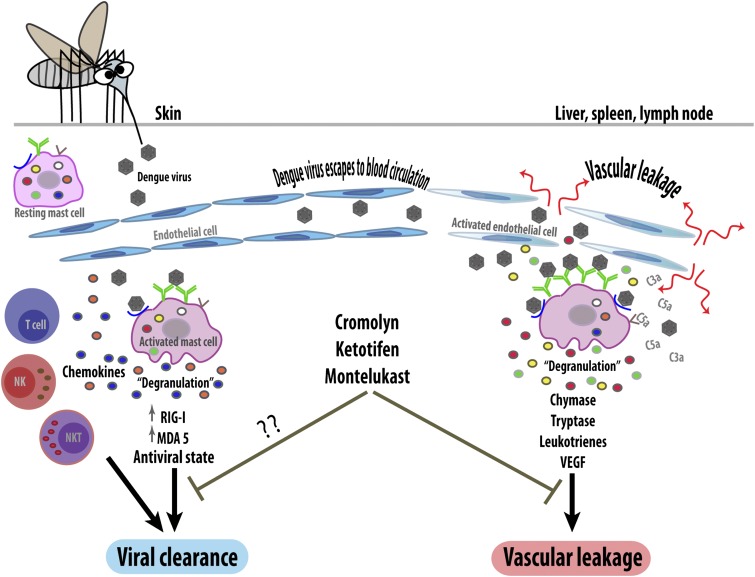
The response of mast cells to dengue virus can be beneficial or detrimental. When a mosquito injects dengue virus (brown hexagons) into the skin, the viruses are detected by specific antibodies (green) or unidentified receptors (blue) on the surface of resting (i.e., non-activated) mast cells. These can then trigger an anti-viral response (left) by releasing the contents of their granules (degranulation) and by upregulating intracellular anti-viral molecules (RIG-I and MDA5). The activated mast cells also secrete signaling molecules called chemokines, which recruit other immune cells including natural killer cells (NK), natural killer T cells (NKT) and T cells, which help to clear the virus. However, if local control mechanisms fail, the virus will enter the bloodstream and be carried to other organs (right). This activates the mast cells in these organs so that they undergo degranulation, releasing ready-made proteases such as chymase and tryptase, and synthesizing inflammatory mediators (leukotrienes and vascular endothelial cell growth factor [VEGF]). These increase the permeability of capillaries, leading to vascular leakage. Mast cells in these organs can also be activated by endogenous inflammatory mediators (such as C3a and C5a) that help the body to remove pathogens. Blocking mast cells (or their mediators) with drugs such as cromolyn, ketotifen and montelukast reduces pathogenic vascular leakage, but might also hamper viral clearance. Anti-mast cell therapy could thus be a double-edged sword.

Medications that block mast cells or their mediators have long been used to treat allergy and asthma, albeit with modest efficacy (Boushey, 2012). However, the use of these drugs in dengue infection must wait until we have a better understanding of how mast cells are regulated. It will be essential to find out how we can block vascular leakage without impeding viral clearance, and achieve overall control of infection without triggering an unwanted immune reaction. Nevertheless, the work of St John et al. offers new hope that it will be possible to treat or prevent DHF using readily available drugs, just as soon as we can identify which patients will benefit, and when.
